# Unraveling the Relationship between Milk Yield and Quality at the Test Day with Rumination Time Recorded by a PLF Technology

**DOI:** 10.3390/ani11061583

**Published:** 2021-05-28

**Authors:** Rosanna Marino, Francesca Petrera, Marisanna Speroni, Teresa Rutigliano, Andrea Galli, Fabio Abeni

**Affiliations:** 1 Centro di Ricerca Zootecnia e Acquacoltura, Consiglio per la Ricerca in Agricoltura e L’analisi Dell’economia Agraria (CREA), via Lombardo 11, 26900 Lodi, Italy; francesca.petrera@crea.gov.it (F.P.); marisanna.speroni@crea.gov.it (M.S.); rutigliano.teresa@gmail.com (T.R.); a.galli@aral.lom.it (A.G.); fabiopalmiro.abeni@crea.gov.it (F.A.); 2 Associazione Regionale Allevatori Lombardia (ARAL), via Kennedy 30, 26013 Crema, Italy

**Keywords:** precision livestock farming, milk quality, rumination time

## Abstract

**Simple Summary:**

Precision livestock farming, by real time monitoring of dairy cows, has the potential to generate a huge amount of data to be used for farm management purposes, as well as in breeding programs. Daily rumination time (RT) recorded by commercial systems is promising in this context because it may be related to individual milk yield and composition. However, it is necessary to assess the ability of sensor data to be used in a predictive model, but also to evaluate and standardize the correct phenotypes, and how they are related to individual variability rather than from other sources. RT data and milk test day (TD) records collected from 691 cows, monitored for thirteen months, were analyzed for the already mentioned goals and to better characterize the effect of high-, medium- and low-level daily RT on milk yield and composition. Our results showed that “animal” in a farm major contributed to the RT total variability, confirming a possible use in breeding program. The higher RT class reported the best productive performance for milk and each solid yield, in spite of a small reduction in their contents, and appears to be related to a higher degree of saturation in the fatty acid profile.

**Abstract:**

The study aimed to estimate the components of rumination time (RT) variability recorded by a neck collar sensor and the relationship between RT and milk composition. Milk test day (TD) and RT data were collected from 691 cows in three farms. Daily RT data of each animal were averaged for 3, 7, and 10 days preceding the TD date (RT_D_). Variance component analysis of RT_D_, considering the effects of farm, cow, parity, TD date, and lactation phase, showed that a farm, followed by a cow, had major contributions to the total variability. The RT_10_ variable best performed on TD milk yield and quality records across models by a multi-model inference approach and was adopted to study its relationship with milk traits, by linear mixed models, through a 3-level stratification: low (LRT_10_ ≤ 8 h/day), medium (8 h/day < MRT_10_ ≤ 9 h/day), and high (HRT_10_ > 9 h/day) RT. Cows with HRT_10_ had greater milk, fat, protein, casein, and lactose daily yield, and lower fat, protein, casein contents, and fat to protein ratio compared to MRT_10_ and LRT_10_. Higher percentages of saturated fatty acid and lower unsaturated and monounsaturated fatty acid were found in HRT_10_, with respect to LRT_10_ and MRT_10_ observations.

## 1. Introduction

Growing interest in precision livestock farming (PLF) tools for real time monitoring of dairy cows has two possible outcomes: use of PLF data for herd management and for genetic improvement programs [1]. These possible uses must rely on a correct labeling process (i.e., to know the meaning of the obtained data, generally as a proxy of a specific biological marker) and on a reliable acquisition system.

Identifying rumination measures reflecting phenotypes relevant to feed efficiency, cow health, and milk composition would be of great interest for both the management and breeding. Gengler [1] reviewed challenges and opportunities of using sensors to define novel traits for assessing and maximizing the genetic potential of dairy cattle. He pointed out that, in a breeding perspective, less accurate values can be accepted when measurements are repeated on the same animal and across members of the same family. Nevertheless, it is necessary to assess and standardize the correct phenotypes (i.e., the traits) we want to measure and their meaning for our selective goals. Again, for use within a genetic improvement program, it is necessary to assess how they are related to an individual variability rather than to other sources of variability. However, the temporal acquisition (continuous) and the big amount of data based on PLF should be carefully considered because the use of PLF data in a breeding program might be computationally complex.

For the use within farm management, it is necessary to assess the real ability of sensors data to be used in a predictive model: are they really able to give information useful to improve the prediction of an interesting outcome?

Rumination is the natural cow’s behavior, which consists in regurgitation, re-mastication, and re-swallowing of boluses. This mechanical process permits increasing the substrate area for microbial fermentation responsible of the production of volatile fatty acids, ammonia, and proteins [2], resulting in a good share of cow’s energy. Rumination activity in dairy cows is associated with feed intake: particle length of the ration and neutral detergent fiber (NDF) content, especially cellulose and lignin, are related to chewing and rumination activities. Rumination time (RT, number of minutes in a given period of time) is associated to nutritional factors of feeds [3], rumen environment, and animal welfare: RT decreases in acute stress conditions, e.g., heat stress [4,5], during disease, e.g., hypocalcaemia [6], subclinical ketosis (SCK) [7], mastitis or digestive disorders, during physiological changes, e.g., calving and estrus events [8]. Analysis of RT changes could be a good predictive tool for discomfort [9].

The percentages of time cows spent (daily) standing, grazing, chewing, drinking, and feeding are related to animal welfare; RT depression leads to reduction of dry matter intake (DMI), causing a decline in milk yield and quality [5]. Milk composition, especially fat to protein ratio (FPR), urea nitrogen, and ketone bodies concentrations, provides information about energy production, protein concentration in the ration, and possible metabolic imbalances. Negative energy balance (NEB) causes: (i) an increase in milk fat content; (ii) a decrease in milk protein content and, at the same time, (iii) an increase of FPR. Given the relationship between milk yield and lactation stage, the FPR is a more sensitive marker compared to the individual use of fat and protein contents: a positive energy balance is indicated by FPR values between 1.2 and 1.4 [10,11].

Soriani et al. [5] reported a positive association between milk yield and RT, Kaufman et al. [7] noticed the same positive association in early-lactation dairy cows plus a negative association with fat composition and FPR in third lactation cows. Although it is well known that the time spent ruminating affects milk yield and quality, the relationship between RT recorded by commercial systems and test day (TD) milk yield and quality has not been quite examined. Expanding these aspects could allow identifying better farm management practices and strategies to influence and gain the composition of milk. In a recent paper, Andreen et al. [12] explored the relationships between RT and milk fat over multiple TD records analyzing data obtained from two sensors (ear tag vs. neck collar) in two commercial farms.

Trying to understand if RT data recorded continuously for each animal in a farm could be used in breeding programs to improve milk production and quality, this study aimed to dissect the components of RT variability recorded daily by a neck collar sensor. We considered the average daily RT of three time periods (measured during the three, seven and ten days preceding the TD) along with TD to evaluate the effect of different daily RT levels (high, medium, and low) on milk yield and composition.

## 2. Materials and Methods

### 2.1. Farm Characteristic and Data Collection

A total of 691 Italian Friesian lactating cows, reared in three commercial farms in Lombardy region (northwest of Italy), were monitored for RT over thirteen months. All animals were fitted with the same activity and rumination collars on the left side of the neck (SCR Heatime^®^ HR System, Engineers Ltd., Netanya, Israel) for automated health and fertility monitoring. This technology was already validated supporting its utilization in our study [13,14].

During the observation period, individual milk yields were recorded in the a.m. milking session in one month and in the p.m. in the following month, by field officers of the Lombardy Regional Breeders Association (ARAL, Crema, Italy) for daily milk yield estimation (ICAR AT4 method), within routine TD recording schemes. Only cows with a minimum 5 days in milk (DIM) were included. At the same time, individual milk samples were collected and analyzed at the Milk Quality Laboratory of the ARAL for fat, protein, casein, and lactose percentages, and saturated (SFA), unsaturated (UFA), monounsaturated (MUFA), and polyunsaturated (PUFA) fatty acids contents (% of fat), by MIR spectrometry using a MilkoScan^TM^ FT6500 Plus Instrument (Foss, Hillerød, Denmark). Daily lactose, protein, casein, and fat yield (kg/day) and FPR were also calculated. Other traits (i.e., urea nitrogen content, somatic cell count, beta-hydroxybutyrate, and ketone bodies) were not considered in the present study. Test day milk yield and composition, DIM, and parity records of each animal were extracted from local farm software. Data on herd management in the three farms were provided by the owners. All animals were housed in freestall buildings and milked twice a day in conventional milking parlors, equipped with automatic cluster removers and electronic milk meters. Milk produced was destined for cheese-making. Feed management of dairy cows was independent in the three farms although not completely different consisting in a total mixed ration, based on whole corn silage, hay and concentrate.

### 2.2. Database Organization and Data Processing

This study is a part of a larger research for which a relational database was constructed using phpMyAdmin (https://www.phpmyadmin.net (accessed on 19 February 2018)) to connect data from multiple sources. For the purpose of this work, RT, TD, and individual cow information data, related to the observation period, were used. The RT data, summarized as 2-h intervals by the software DataFlow II (SCR Engineers Ltd., Netanya, Israel), were firstly checked for possible missing values. Cows with missing data during the 3, 7, or 10 days previous TD were removed. New variables for RTs were created and based on the average daily RT of the 3, 7, and 10 days before TD: a unique RT_D_ value (RT_3_, RT_7_, RT_10_) for each cow at each TD was calculated; particularly, the 12 data points of RT within day were summed to obtain the daily RT and then they were averaged across days (3, 7, and 10 days preceding each TD).

### 2.3. Statistical Analysis

Descriptive statistics for DIM at the TD, RT_D_ (average of the 3, 7, and 10 days before TD), TD milk production, and composition (8 TD traits) data were calculated; they were also summarized within farms and TD date. As reported in Andreen et al. [12], to assess how RT differs within each observation period (3, 7, and 10 days) at the TD, standard deviations (SD) of RT_D_ for each cow were calculated. Mean values of SD for RT_D_ were also computed within the three farms and for the complete dataset. 

Variance component analysis (VCA) was performed on the whole dataset to assess how the variability of the RT is structured considering the effects of the farm, cow, number of lactations, date of TD, and DIM. Variance component (VC) contributions to the total variability, predicted by application of REML estimation, and confidence intervals (CI) of VCs were estimated using the VCA R package. The VCA was also applied to the three RT_D_ in order to assess the variance partition between cow, number of lactations, date of TD, DIM, and residuals in each farm.

Starting from the hypothesis that models considering the three different RT variables (RT_3_, RT_7_, RT_10_) could fit the TD milk production and composition variables equally well, we performed multi-model inference (MMI) using the dredge function from the MuMIn package [15] for an explorative model selection and to identify the interval of days for which RT best fits the milk traits. In this way, we calculated the relative variable importance of RT_3_, RT_7_, and RT_10_ using the importance function in MuMIn [15].

The model tested for each dependent variable was:

Y = µ + RT_D_ + A + F + D + ε, (1)

where Y is the vector of observations of the dependent variable (milk yield (kg/day); lactose, protein, casein and fat contents and daily yields (as % and kg/day)); µ is the overall mean; RT_D_ is the fixed effect of the three RT_D_ periods (RT_3_, RT_7_, RT_10_), A, F, and D, are, respectively, the random effect of the animal, farm, and date of TD; ε is the residual error. The mixed effect linear model was fitted using lm4 package [16]. The relative variable importance scores for each RT_D_ were calculated (summing the Akaike weights of the candidate models in which that variable was present) and ranked.

The results obtained by the model selection analysis were considered in the subsequent step aimed to evaluate the contribution of RT on TD milk records. Therefore, the RT_D_ with the highest important score for the most of the studied variables (RT_10,_ as shown later in the results) was retained.

Hence, the contribution of the RT_10_ on TD milk records, such as yield and composition, was evaluated by linear mixed models using lme4 R package [16] (R Version 3.4.4., CRAN Garr Mirror, Milano, Italy) adding in the model the effects of the different lactation stage, parity, and farms.

The estimates were fitted using the REML method. 

The model was built as:

Y = µ + RT_10L_ + F + P + L + D + C + ε, (2)

where Y is the vector of observations of the dependent variable (daily milk yield (kg/day); lactose, protein, casein, and fat contents and daily yields (as % and kg/day); saturated and unsaturated fatty acid (as % of fat); mono- and polyunsaturated fatty acid (as % of fat); FPR); µ is the overall mean; RT_10L_ is the fixed effect of the animal daily RT calculated on the 10 days preceding the TD and stratified in three levels, low (LRT_10_ ≤ 8 h/day), medium (8 h/day < MRT_10_ ≤ 9 h/day), and high RT_10_ (HRT_10_ > 9 h/day); F is the fixed effect of the farm; P is the fixed effect of parity (three levels: 1, 2, and ≥3 calvings); L is the fixed effect of lactation phase (three levels: ≤60, 61–180 and ≥181 DIM); D is the fixed effect of TD date; C is the random effect of each animal nested within the rumination group level (1 + R_10L_|cow); and ε is the residual error. The best model for each dependent variable was retained according to the minimum Akaike’s information criterion. Least square means (LSM) and 95% CI were calculated for TD milk yield and composition records by the three levels of RT using the lsmeans R package. The degrees-of-freedom were estimated with the Kenward–Roger method and the adjusted *p* values of contrasts were computed with the Tukey method. Model assumptions were verified by plotting residuals versus fitted values.

## 3. Results

### 3.1. General Dataset Information

Descriptive statistics for DIM, daily RT_D_ (calculated as the average of RT over a period of 3-, 7-, and 10-days preceding TD date) and milk yield and composition traits at the TD for the entire dataset records (*n* = 3451) were reported in Table 1.

The proximity between median and mean and the similar distance between the median and their nearest quartiles for the three RT variables suggests an almost symmetric shape of our observations around the central values. This was not mirrored in the distributions of the considered dependent milk yield and quality items, suggesting a different extent of RT effect towards its higher or lower values.

In Table 2, results were presented within each farm and the number of observations were 756 for farm 1, 510 for farm 2, and 2185 for farm 3.

The same descriptive statistics at the farm level suggest some differences in the lactating herd composition for DIM, but at the same time confirm how 75% of the cow population fell in the range of medium-to-high yielding dairy cows in each farm. The three herds were comparable in milk productions and composition along the period of the experimentation. These data were reported in Table S1, in which summary statistics were shown by TD date and farms. Average RT was mainly similar between farms 1 and 3 for each of the three periods while farm 2 always had a larger value (Table 2). 

**Figure 1:**
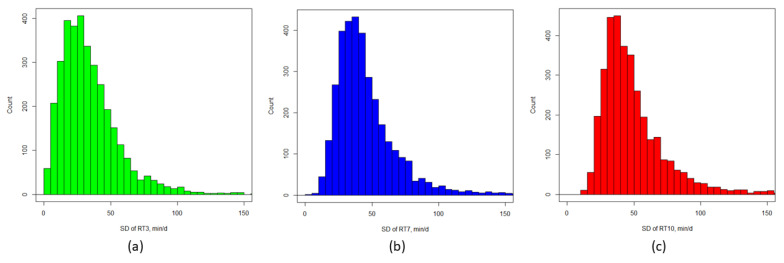
Histogram of standard deviation (SD) of rumination time (RT), calculated for each cow considering a period of: (**a**) three (RT_3_), (**b**) seven (RT_7_), and (**c**) ten (RT_10_) days preceding each test day date. Rumination time was measured with a neck collar (SCR Heatime^®^ HR System, Engineers Ltd., Netanya, Israel). In the figure min/d = minute/day.

Daily RT for each cow during the three periods of observation varied considerably, in fact, the mean SD of RT was 34 ± 23 min for RT_3_, 46 ± 25 min for RT_7_, and 50 ± 26 min for RT_10_. The majority of cows had an SD of RT in the days before TD greater than 20 min, with most falling between 30 and 60 min as shown in Figure 1. 

In Supplementary Table S2 we presented the mean SD of RT_D_ (3-, 7-, and 10-days preceding TD date) within farm 1, 2, and 3.

The contribution of each random effect (farm, cow, parity, DIM, date of TD) to the variance of the dependent variable RT_D_ from VCA was presented in Table 3.

### 3.2. Importance of Rumination Time Intervals

Three different periods (3, 7, and 10 days) of daily RTs were analyzed using MMI to assign their relative variable importance on the daily milk production (daily yield) and on the principal parameters of composition (lactose, protein, casein, and fat contents). The output of MMI analysis is presented in the Table 4; the variable importance is a value between 0 and 1, where 1 was assigned to the variable found in all the best models ranked on the AIC value. 

In model 1, the RT periods were found to be important in the following order: RT_10_ ≥ RT_3_ > RT_7_ for most of the variables, except for protein and lactose contents (RT_10_ > RT_7_ > RT_3_) and fat daily yield (RT_7_ > RT_10_ > RT_3_).

### 3.3. Mixed Model Analysis

Model 2 was designed to test the effects of RT_10_ level (high, medium, and low), including parity, farm, lactation phase, TD date as predictors, on milk traits, by fitting the RT_10_ differences between individual cows as a random term and to estimate the between-animals variance in the trait of interest. Results of analysis of variance of fixed effects are reported in Table 5.

All considered fixed effects were significant (*p* < 0.05) in explaining the variability of milk traits, except parity for FPR and polyunsaturated fatty acid content, farm for unsaturated, and monounsaturated fatty acid content, and RT_10L_ for polyunsaturated fatty acid content.

Least square means (LSM) of milk traits by the three levels of RT and 95% CI are reported in Table 6.

Cows with HRT_10_ had significantly greater (*p* < 0.0001) daily milk yield (38.29 vs. 35.80 and 31.99 kg/day;), protein (1.28 vs. 1.22 and 1.10 kg/day), and casein daily yield (0.99 vs. 0.94 and 0.85 kg/day), lactose daily yield and percentage (1.88 vs. 1.74 and 1.54 kg/day; 4.90 vs. 4.86 and 4.81%), and lower fat percentage (3.88 vs. 4.07 and 4.30%) compared to ones with MRT_10_ and LRT_10_. Cows in the group HRT_10_ showed also significantly (*p* < 0.0001) higher fat daily yield (1.47 vs. 1.36 kg/day), while lower FPR (1.15 vs. 1.24), protein (3.38 vs. 3.48%) and casein percentages (2.62 vs. 2.69%) compared to LRT_10_. Analysis of fatty acid composition (% on fat) showed significant differences between HRT_10_ and LRT_10_ (*p* < 0.0001) and between MRT_10_ and LRT_10_ groups (*p* < 0.05). Percentages of saturated fatty acid were slightly higher (66.48 vs. 65.40 and 66.03%), while unsaturated (29.83 vs. 31.44 and 30.51%) and monounsaturated fatty acid (24.91 vs. 26.08 and 25.38%) were lower in HRT_10_, with respect to LRT_10_ and MRT_10_ groups. Non-significant differences between classes were found for polyunsaturated fatty acid percentages.

## 4. Discussion

The PLF technology employed in our study was also reported in several research papers, from around the world, in the past decade [4,5,7,8,9,12,13,14,17], and was validated and widely adopted for estrus detection [17]. We reported our first observations to monitor heat stress by RT reduction during summer 2015 [4]. As we mentioned in the descriptive statistics, RT exhibited slight differences among the farms; those differences, however, seem attributable to specific farm effects because the technology (and the component generation within the technology) was the same. The values of RT recorded within our study agree with those reported by DeVries et al. [18]. The mean value SD for RT_7_ (46 ± 25 min) was similar to the 48 ± 23 min reported in Andreen et al. [12] and recorded over a 7 days period preceding TD. They also reported a distribution a SD of RT in the 7 days before TD, with most falling between 30 and 60 min.

### 4.1. Time–Phenotype Meaning Assessment

The possible identification of a new phenotype and its meaning needs to be looked at. Before we consider data or a sum of them as a new phenotype, it is necessary to assess the relative variance due to the subject (cow) within a model and its ratio to the day-by-day variance, as well as to other possible sources of variance. Moretti et al. [9] reported a variance explained by the animal of at least more than 12% within their models, and with a ratio of more than 6 with the day-by-day variance. From our previous observations during summer season, the variance in daily RT due to the cow was more than 33% of the total variance, and equal to that due to the day effect [4]. Byskov et al. [19] found that 48% of the total variation was due to individual variations between cows and 32% was accounted for feed intake. Byskov et al. [20] collected their data with the same device of our study and, for the statistical analysis, they used the weekly average of daily RT for each cow.

In the current study, we first analyzed the variance partition of RT to assess the cow effect at 3, 7, and 10 days before milk test day, to understand the possible role of between-subject variability in rumination, to determine the effect on milk yield and its components. This was conducted considering previous findings that evidenced a different variance partition, taking into account daytime RT and nighttime RT separately [4]. The range of cow effect was quite similar for each average RT period, with a minimum value (20.3%) for the 10 days model, and a maximum (20.9%) for the 7 days model. This is an intermediate value between our previous finding during summer heat stress (33.9 and 54.8% for total daily and for nighttime only RT calculated within farm [4]), where the RT was from a single day, and the value of 12.33% reported by Moretti et al. [9]. As reported by Egger-Danner et al. [21], rumen activity may be a possible indicator trait for feed efficiency as well as a predictor of: milk quality [12]; possible metabolic disorders, such as subclinical ketosis [22] or sub-acute ruminal acidosis [23], and other health problems in dairy cows [9].

The time-relationship between a signal from RT and the predictable outcome seems reasonably affected by the event nature itself. Moretti et al. [9], using the AIC and the Pseudo-R^2^ as criteria to select the best model predicting different problems, evidenced different window’s size according to each problem: 5 days for generic disease; 3 days for reproductive diseases; 1 day for mastitis; 5 days for locomotor issues; and a window’s size of 1 day for gastroenteric diseases. No specific relationships were reported on milk features. Stangaferro et al. [24] studied the lag between the first drop of a combined health index (HIS, derived from the instrument recording activity and RT) and the day of a clinical diagnosis (CD); they reported −3, −1.6, −0.5, and −2.1 days for displaced abomasum, ketosis, indigestion, and all metabolic and digestive disorders, respectively. In light of those results, it seemed reasonable to test the RT averaged for three different time periods to assess which of them may affect milk yield and quality.

From this point of view, our results are quite original. Our aim was comparable with those leading Andreen et al. [12] to detect a possible relationship between rumination activity and milk quality; however, they considered only the results attained during the 7 days before milk test. In our comparison among 3, 7, and 10 days mean rumination time before milk test, we found the best prediction performance using 10 days, followed by the 3 days mean. Our approach with the multi-model inference for the selection of the best averaged RT was due to the purpose to assess which is the average RT (the trait) that, changing the model, still maintains the best relationship with the milk yield and composition variables.

### 4.2. Rumination Time and TD Milk Yield

The proportional increase in milk yield in the groups with higher rumination times agrees with the results recorded by Soriani et al. [5] and Johnston and DeVries [25]. The extent of increased milk yield with RT was similar to that recorded by Johnston and DeVries [25]; they predicted an increase in daily milk yield by 1.26 kg/day for every hour increase in RT per day. Their results applied to maximum and minimum RT observation in our study could predict 8.7 kg/day difference in milk yield between them. An important question rises about the explanation for this improved milk yield. The options are essentially two: an increased DMI or an increased feed efficiency.

The relationships among DMI, RT, and milk yield are not yet clarified [26]. Generally, the great share of results analyzed by Beauchemin [26] support a positive (from weak to moderate, depending also from the parity) relationship between RT and milk yield. However, the extent of the recorded performance in milk yield did not seem attributable to a supposed increase in DMI. As evidenced by Clément et al. [27], the lack of a significant contribution from the inclusion of RT within the DMI predictive equation by NRC [28] did not support the link among increased RT and a possible DMI increase to fully explain our results on milk yield. Therefore, our results on milk yield may be also the consequence of an improved rumen activity.

Schirmann et al. [29] found that RT can be used to estimate intra-cow variation in feeding behavior and intake, but daily summaries of rumination behavior are a poor indicator of DMI. In fact, they observed that following 2 h-periods of high feeding times and intakes, 42 dry cows spent more time ruminating. This relationship peaked approximately 4 h after feeding. However, cows that spent more time ruminating per day spent less time feeding and rumination times were not related to DMI.

Results from a recent paper reported an important relationship between RT increase just after calving and the attainment of high performance in the started lactation [30]. The researchers did not report DMI; however, the relationship between both the change in RT day-by-day, and the daily RT with milk yield suggested the goodness of RT as a behavioral marker of the digestive ability to support the rise in yielding performance at the beginning of lactation, as well as a possible predictive tool for the overall lactation.

### 4.3. Rumination Time and TD Milk Components

Milk components, namely fat, are affected by the rumen activity. DeVries et al. [18] pointed out the close relationship between total daily RT and the risk to develop subacute ruminal acidosis (SARA). In fact, RT appears as a good marker of the cow’s ability to cope with a SARA challenge.

The relationship between daily RT and milk components may be driven by two factors: a simple dilution effect due to a different milk yield; different availability of precursors at the udder level that derive from rumen activity and absorption. In the current study, it is not easy to separate these possible effects. However, the higher milk yield related to the higher RT_10_ suggests a main role of the former factor. This was also confirmed by the increase in fat yield in higher ruminating cows.

The overall reduction in milk fat content in our study agrees with the results reported by Kaufman et al. [7] for third lactation cows. Looking at the results from Salfer et al. [31], there is concordance between increased RT and a following increase of rumen pH throughout the day. We suppose that increased RT may be associated to a rumen environment favorable for acetic and butyric producing microflora, leading to an increased availability of milk fat precursors absorbed, and then available at the udder level.

An important question arises from the higher level of SFA in milk fat from higher ruminating cows. There are no specific papers to refer about a direct relationship between RT and SFA; however, Toledo-Alvarado et al. [32] reported a significant decrease of SFA as a % of total fatty acid (FA) at the time of estrus, the same time we know to be characterized by a decrease in RT [17,33]. At the same time, the results reported by DeVries et al. [18] and those reported by Alzahal et al. [34], where a situation prone to subacute ruminal acidosis evidenced the effect on milk FA profile, suggest how higher RT may be favorable to an increase in milk SFA. To our knowledge, no other papers are available to support the possible time relationship between RT and SFA content in milk.

The possible dilution effect with increasing milk yield was confirmed for milk protein content, with lower percentage in the milk of cows with higher RT, and this agrees with the findings by Johnston and DeVries [25]. According to [25], protein yield increased with RT_10_ levels. This was the combined result from the increased milk yield, even with a reduced milk protein content due to a dilution effect, but, therefore, without rumen synthesis impairment.

Contrarily, results on solid milk yield evidenced leading action by the increased milk yield as a consequence from increased RT. Johnston and DeVries [25] also reported an increased milk protein yield associated to increased RT.

## 5. Conclusions

Development of sensors for estrus detection rapidly evolved, providing new opportunities to monitor cow welfare and support herd management; moreover, technologies can support farmers in managing animal-based differences in cow adaptations to changing environments. These results are an attempt to identify a possible phenotype (RT and its timing) that may be useful for applications in the study of its relationships with milk yield and quality. A 10-day period before the TD date seems to be the best choice to analyze the effects of RT on milk yield and quality.

The higher RT class reported the best productive performance for milk and for each solid yield, in spite of a small reduction in their contents. The highest RT seems related to the highest degree of saturation in the FA profile.

From these preliminary results, we are encouraged to further study RT as a marker of rumen activities affecting milk yield and quality at an individual level.

## Figures and Tables

**Table 1:** Descriptive summary statistics of the entire dataset.

Variable	Mean ± SD	1st Quartile	Median	3rd Quartile
DIM (at the TD)	163.16 ± 108.18	74.00	148.00	238.00
Rumination Time (min/day)				
RT_3_	508.23 ± 88.88	455.33	511.00	564.67
RT_7_	508.56 ± 85.19	458.43	510.57	563.36
RT_10_	508.27 ± 84.01	457.45	508.80	560.40
TD milk production and composition records				
Milk yield, kg/day	35.33 ± 9.53	28.90	34.60	41.10
Protein, %	3.48 ± 0.39	3.21	3.45	3.72
Protein, kg/day	1.21 ± 0.28	1.02	1.21	1.39
Casein, %	2.71 ± 0.32	2.49	2.69	2.91
Casein, kg/day	0.94 ± 0.22	0.80	0.94	1.09
Fat, %	4.08 ± 1.03	3.45	4.01	4.62
Fat, kg/day	1.42 ± 0.48	1.11	1.35	1.64
Lactose, %	4.88 ± 0.23	4.77	4.91	5.03
Lactose, kg/day	1.73 ± 0.48	1.40	1.70	2.03

RT_3_, RT_7_, RT_10_, are the three average rumination time in min/day calculated at the periods of 3, 7, and 10 days preceding the test day. SD = standard deviation; DIM = days in milk; TD = milk test day; RT = rumination time.

**Table 2:** Descriptive summary statistics of the three farms.

Variable	Farm	Mean ± SD	1st Quartile	Median	3rd Quartile
DIM (at the TD)	Farm-1	125.55 ± 91.50	53.75	107.00	177.00
	Farm-2	196.17 ± 120.51	99.25	188.00	272.50
	Farm-3	168.47 ± 106.94	79.00	158.00	245.00
Rumination Time (min/day)					
RT_3_	Farm-1	482.26 ± 81.30	434.92	490.33	535.75
	Farm-2	617.34 ± 64.94	580.42	619.17	660.67
	Farm-3	491.74 ± 76.97	448.33	498.67	542.67
RT_7_	Farm-1	488.11 ± 76.39	449.75	496.00	539.00
	Farm-2	617.65 ± 60.54	580.75	618.00	656.50
	Farm-3	490.17 ± 73.01	448.71	496.57	541.43
RT_10_	Farm-1	489.47 ± 74.09	452.38	499.60	538.70
	Farm-2	618.02 ± 58.49	582.43	619.55	654.93
	Farm-3	489.17 ± 71.78	447.10	493.60	540.00
TD milk production and composition records					
Milk yield, kg/day	Farm-1	34.57 ± 7.86	29.40	34.45	39.70
	Farm-2	36.42 ± 9.25	30.40	35.50	42.58
	Farm-3	35.34 ± 10.08	28.30	34.50	41.50
Protein, kg/day	Farm-1	1.17 ± 0.24	1.01	1.17	1.33
	Farm-2	1.24 ± 0.24	1.07	1.24	1.41
	Farm-3	1.22 ± 0.30	1.01	1.21	1.41
Casein, kg/day	Farm-1	0.90 ± 0.19	0.78	0.91	1.03
	Farm-2	0.96 ± 0.19	0.84	0.96	1.09
	Farm-3	0.95 ± 0.23	0.80	0.95	1.10
Fat, kg/day	Farm-1	1.32 ± 0.31	1.11	1.31	1.53
	Farm-2	1.47 ± 0.46	1.18	1.40	1.68
	Farm-3	1.44 ± 0.53	1.10	1.35	1.69
Lactose, kg/day	Farm-1	1.71 ± 0.40	1.45	1.70	1.97
	Farm-2	1.75 ± 0.46	1.43	1.73	2.05
	Farm-3	1.73 ± 0.51	1.37	1.69	2.05

RT_3_, RT_7_, RT_10_, are the three average rumination time in min/day calculated at the periods of 3, 7, and 10 days preceding the test day. SD = standard deviation; DIM = days in milk; TD = milk test day; RT = rumination time.

**Table 3:** Variance components (VCs) of rumination time at three (RT_3_), seven (RT_7_), and ten (RT_10_) days preceding each test day, partitioned among the random effect of farm, the random effect of cow, the random effect of parity, the random effect of days in milk, the random effect of test day date and the residual error of observations. VCs were also reported within the three farms.

Item	Farm	Cow	Parity	DIM	TD Date	Residual Error	Total
RT_3_	5227 (46.3%)	2322 (20.6%)	17 (0.2%)	250 (2.2%)	268 (2.4%)	3203 (28.4%)	11,287
RT_7_	5186 (49.2%)	2201 (20.9%)	12 (0.1%)	377 (3.6%)	262 (2.5%)	2497 (23.7%)	10,534
RT_10_	5440 (51.9%)	2132 (20.3%)	9 (0.1%)	456 (4.4%)	191 (1.8%)	2258 (21.5%)	10,486
RT_3_	Farm-1	1925 (28.5%)	61 (0.9%)	396 (5.9%)	427 (6.3%)	3952 (58.5%)	6761
RT_7_	Farm-1	2086 (34.9%)	18 (0.3%)	423 (7.1%)	498 (8.3%)	2954 (49.4%)	5978
RT_10_	Farm-1	2054 (36.7%)	26 (0.5%)	583 (10.4%)	340 (6.1%)	2590 (46.3%)	5593
RT_3_	Farm-2	3452 (58.4%)	598 (10.1%)	285 (4.8%)	172 (2.9%)	1403 (23.7%)	5911
RT_7_	Farm-2	3299 (60.9%)	730 (13.5%)	108 (2.0%)	220 (4.1%)	1058 (19.5%)	5415
RT_10_	Farm-2	3370 (61.8%)	881 (16.2%)	0 (0.0%)	228 (4.2%)	973 (17.8%)	5451
RT_3_	Farm-3	2432 (39.2%)	11 (0.2%)	298 (4.8%)	236 (3.8%)	3234 (52.1%)	6211
RT_7_	Farm-3	2235 (40.6%)	4 (0.1%)	432 (7.9%)	216 (3.9%)	2618 (47.6%)	5505
RT_10_	Farm-3	2167 (41.1%)	0 (0.0%)	506 (9.6%)	204 (3.9%)	2402 (45.5%)	5279

**Table 4:** Variable importance of rumination time at the three observation periods on test day records.

Item	RT_3_	RT_7_	RT_10_
Milk yield, kg/day	1.00	0.79	1.00
Protein, kg/day	1.00	0.43	1.00
Protein, %	0.35	0.52	1.00
Casein, kg/day	1.00	0.49	1.00
Casein, %	0.44	0.38	1.00
Fat, kg/day	0.34	0.68	0.59
Fat, %	1.00	0.96	1.00
Lactose, kg/day	1.00	0.91	1.00
Lactose, %	0.33	0.54	1.00

Variable importance is based on multi-model inference (MMI) and it ranged from 0.0 (least important) to 1.0 (most important); RT_3_, RT_7_, RT_10_, are the three average rumination time in min/day calculated at the periods of 3, 7, and 10 days preceding the test day.

**Table 5:** ANOVA test for fixed effects of model 2.

Item	RT_10L_	F	P	L	D
Milk yield (kg/day)	2.76 × 10^−44^ ***	1.72 × 10^−3^ **	1.13 × 10^−48^ ***	9.23 × 10^−186^ ***	1.29 × 10^−26^ ***
Fat (%)	3.73 × 10^−17^ ***	3.88 × 10^−15^ ***	2.13 × 10^−04^ ***	9.49 × 10^−22^ ***	9.40 × 10^−187^ ***
Fat (kg/day)	7.97 × 10^−7^ ***	2.69 × 10^−21^ ***	7.93 × 10^−20^ ***	3.94 × 10^−82^ ***	9.10 × 10^−156^ ***
Protein (%)	3.63 × 10^−8^ ***	3.02 × 10^−2^ *	4.19 × 10^−20^ ***	6.20 × 10^−219^ ***	1.83 × 10^−111^ ***
Protein (kg/day)	2.08 × 10^−39^ ***	3.14 × 10^−6^ ***	1.86 × 10^−38^ ***	2.02 × 10^−91^ ***	2.34 × 10^−51^ ***
FPR	1.28 × 10^−10^ ***	1.91 × 10^−12^ ***	8.91 × 10^−1^	8.31 × 10^−52^ ***	1.45 × 10^−174^ ***
Lactose (%)	1.05 × 10^−14^ ***	9.17 × 10^−5^ ***	4.09 × 10^−49^ ***	3.79 × 10^−30^ ***	7.73 × 10^−30^ ***
Lactose (kg/day)	4.18 × 10^−47^ ***	2.13 × 10^−3^ **	1.34 × 10^−32^ ***	8.60 × 10^−186^ ***	1.26 × 10^−28^ ***
Casein (%)	8.72 × 10^−07^ ***	9.39 × 10^−6^ ***	6.98 × 10^−27^ ***	7.65 × 10^−229^ ***	4.08 × 10^−83^ ***
Casein (kg/day)	2.20 × 10^−39^ ***	7.94 × 10^−9^ ***	7.57 × 10^−34^ ***	2.37 × 10^−86^ ***	9.94 × 10^−46^ ***
SFA (%)	6.85 × 10^−7^ ***	8.50 × 10^−7^ ***	1.73 × 10^−4^ ***	3.57 × 10^−95^ ***	0.00 ***
UFA (%)	1.42 × 10^−10^ ***	8.84 × 10^−2^ .	3.53 × 10^−2^ *	3.86 × 10^−104^ ***	4.50 × 10^−296^ ***
MUFA (%)	1.80 × 10^−6^ ***	2.97 × 10^−1^	1.25 × 10^−7^ ***	2.53 × 10^−59^ ***	0.00 ***
PUFA (%)	7.47 × 10^−2^ .	8.01 × 10^−4^ ***	2.82 × 10^−1^	3.72 × 10^−9^ ***	3.99 × 10^−194^ ***

Significance codes: ‘.’ *p* < 0.1, ‘*’ *p* < 0.05, ‘**’ *p* < 0.01, ‘***’ *p* < 0.001; RT_10L_ = is the fixed effect of the animal daily rumination time calculated on the 10 days preceding the test day and stratified in three levels, low (LRT_10_ ≤ 8 h/day), medium (8 h/day < MRT_10_ ≤ 9 h/day), and high RT_10_ (HRT_10_ > 9 h/day); F = is the fixed effect of the farm (three levels); P = is the fixed effect of parity (three levels: 1, 2 and ≥3 calvings); L = is the fi×ed effect of lactation phase (three levels of days in milk: DIM ≤ 60, 61–180 DIM, ≥181 DIM); D = is fi×ed effect of test day date. FPR = fat to protein ratio; SFA = saturated fatty acid; UFA = unsaturated fatty acid; MUFA = monounsaturated fatty acid; PUFA = polyunsaturated fatty acid.

**Table 6:** Least square means, standard error and 95% CI in the three RT_10_ groups.

Item	Low RT_10_ Mean ± SE (95% CI)	Medium RT_10_ Mean ± SE (95% CI)	High RT_10_ Mean ± SE (95% CI)
Milk yield (kg/day)	31.99 ^a^ ± 0.38 (31.24–32.75)	35.80 ^b^ ± 0.36 (35.10–36.50)	38.29 ^c^ ± 0.35 (37.60–39.98)
Fat content (%)	4.30 ^c^ ± 0.04 (4.21–4.38)	4.07 ^b^ ± 0.04 (3.99–4.15)	3.88 ^a^ ± 0.04 (3.81–3.95)
Fat yield (kg/day)	1.36 ^a^ ± 0.02 (1.33–1.40)	1.44 ^b^ ± 0.02 (1.40–1.48)	1.47 ^b^ ± 0.02 (1.44–1.50)
Protein content (%)	3.48 ^c^ ± 0.02 (3.44–3.51)	3.43 ^b^ ± 0.02 (3.40–3.46)	3.38 ^a^ ± 0.01 (3.35–3.41)
Protein yield (kg/day)	1.10 ^a^ ± 0.01 (1.07–1.12)	1.22 ^b^ ± 0.01 (1.20–1.24)	1.28 ^c^ ± 0.01 (1.26–1.30)
Fat to protein ratio	1.24 ^c^ ± 0.01 (1.22–1.26)	1.19 ^b^ ± 0.01 (1.17–1.21)	1.15 ^a^ ± 0.01 (1.13–1.17)
Lactose content (%)	4.81 ^a^ ± 0.01 (4.79–4.83)	4.86 ^b^ ± 0.01 (4.84–4.88)	4.90 ^c^ ± 0.01 (4.88–4.91)
Lactose yield (kg/day)	1.54 ^a^ ± 0.02 (1.50–1.58)	1.74 ^b^ ± 0.02 (1.71–1.78)	1.88 ^c^ ± 0.02 (1.84–1.91)
Casein content (%)	2.69 ^b^ ± 0.01 (2.67–2.72)	2.66 ^b^ ± 0.01 (2.64–2.69)	2.62 ^a^ ± 0.01 (2.60–2.65)
Casein yield (kg/day)	0.85 ^a^ ± 0.01 (0.83–0.87)	0.94 ^b^ ± 0.01 (0.93–0.96)	0.99 ^c^ ± 0.01 (0.98–1.01)
SFA (% of fat)	65.41 ^a^ ± 0.18 (65.04–65.77)	66.03 ^b^ ± 0.16 (65.71–66.35)	66.48 ^c^ ± 0.15 (66.18–66.78)
UFA (% of fat)	31.44 ^c^ ± 0.21 (31.03–31.85)	30.51 ^b^ ± 0.18 (30.15–30.87)	29.83 ^a^ ± 0.17 (29.50–30.17)
MUFA (% of fat)	26.08 ^c^ ± 0.2 (25.69–26.47)	25.38 ^b^ ± 0.17 (25.03–25.72)	24.91 ^a^ ± 0.16 (24.59–25.23)
PUFA (% of fat)	3.96 ^a^ ± 0.03 (3.90–4.03)	3.99 ^a^ ± 0.03 (3.92–4.06)	4.05 ^a^ ± 0.03 (3.99–4.11)

^a, b, c^: different superscripts within a row indicate differences (*p* < 0.05) between means based on mean separation with the LSD test. RT_10_ = average rumination time in min/day calculated at the period of 10 days preceding the test day. Low RT_10_ = cows with a daily RT_10_ ≤ 8 h/day; Medium RT_10_ = cows with a daily RT_10_ between 8 and 9 h/day; High RT_10_ = cows with a daily RT_10_ > 9 h/day. SFA = saturated fatty acid; UFA = unsaturated fatty acid; MUFA = monounsaturated fatty acid; PUFA = polyunsaturated fatty acid.

## Data Availability

Restrictions apply to the availability of these data. Data were obtained within a private agreement between CREA and SCR Europe and are available from the authors only with the permission of SCR Europe.

## Supplementary Materials

The following are available online at https://www.mdpi.com/article/10.3390/ani11061583/s1, Table S1: Descriptive summary statistic within the three farms at each test day, Table S2: Standard deviations of RT_D_ in the three farms.
