# Supplementary material for: Unraveling the Relationship between Milk Yield and Quality at the Test Day with Rumination Time Recorded by a PLF Technology
Source: Animals (Basel). 2021 May 28;11(6):1583. doi: 10.3390/ani11061583 (PMC8228303; doi:10.3390/ani11061583)
Supplement: Supplementary file 1 [file animals-11-01583-s001.zip › supplementary table S2.pdf]

Table S2. SD of RT_D in the three farms

Variable	Farm	Mean		SD	1st quartile	Median	3rd quartile
SD RT_3 (min/d)	Farm-1	37.74	±	24.12	21.73	32.70	47.08
	Farm-2	30.36	±	18.36	17.21	28.14	39.69
	Farm-3	34.10	±	23.17	18.57	29.17	44.21
SD RT_7 (min/d)	Farm-1	49.87	±	24.25	33.53	44.16	59.66
	Farm-2	39.36	±	21.04	27.25	36.01	44.84
	Farm-3	46.18	±	25.58	29.76	40.14	54.68
SD RT_{10} (min/d)	Farm-1	54.88	±	25.39	37.92	49.16	65.53
	Farm-2	41.41	±	20.02	29.82	36.95	46.70
	Farm-3	50.06	±	26.78	32.72	43.34	58.39

RT_3, RT_7, RT_{10}, are the three average rumination time in min/d calculated at the periods of 3, 7 and 10 days preceding the test day
